# Wax Confinement with Carbon Nanotubes for Phase Changing Epoxy Blends

**DOI:** 10.3390/polym9090405

**Published:** 2017-08-31

**Authors:** Giulia Fredi, Andrea Dorigato, Luca Fambri, Alessandro Pegoretti

**Affiliations:** 1 Department of Industrial Engineering, University of Trento, via Sommarive 9, 38123 Trento, Italy; giulia.fredi@unitn.it (G.F.); andrea.dorigato@unitn.it (A.D.); luca.fambri@unitn.it (L.F.); 2 National Interuniversity Consortium for Science and Technology of Materials (INSTM), Via G. Giusti 9, 50121 Firenze, Italy

**Keywords:** multifunctional composites, carbon nanotubes, epoxy resin, thermal energy storage, phase change materials

## Abstract

A paraffin wax was shape stabilized with 10 wt % of carbon nanotubes (CNTs) and dispersed in various concentrations in an epoxy resin to develop a novel blend with thermal energy storage capabilities. Thermogravimetric analysis showed that CNTs improve the thermal stability of paraffin, while differential scanning calorimetry showed that the paraffin kept its ability to melt and crystallize, with enthalpy values almost proportional to the paraffin fraction. In contrast, a noticeable loss of enthalpy was observed for epoxy/wax blends without CNTs, which was mainly attributed to the partial exudation of paraffin out of the epoxy matrix during the curing phase. Dynamic mechanical thermal analysis contributed to elucidate the effects of the melting of the paraffin phase on the viscoelastic properties of the epoxy blends. Flexural elastic modulus and strength of the blends decreased with the wax/CNT content according to a rule of mixtures, while flexural strain at break values deviate positively from it. These results show the potentialities of the investigated epoxy blends for the development of multifunctional structural composites.

## 1. Introduction

Thermal energy storage (TES) consists in storing excess heat and releasing it where and when needed, thus filling the gap between thermal energy demand and supply. Accumulating and releasing latent thermal energy of organic phase change materials (PCMs) is particularly effective, as these materials can store a large amount of heat per unit mass over a narrow temperature range, with limited volume variations [1,2]. The most widely used organic PCMs are paraffin waxes, poly(ethylene glycol), and fatty acids [3]. In spite of their advantages over other PCMs, they all display confinement problems when heated above their melting temperature and a relatively low thermal conductivity [4]. The first drawback can be overcome by encapsulating PCMs in polymeric or inorganic shells [5], by embedding them in a polymer network [6] or by confining them in porous carbonaceous or inorganic structures, thus forming a shape-stabilized PCM (SS PCM) [7,8]. If the shape stabilization is performed with a thermally conductive structure, such as a carbonaceous nanofiller, the problem of the low thermal conductivity can also be partially reduced and the overall thermal exchange improved [2,9,10].

TES systems based on PCMs find applications in several fields. For example, they can be integrated into walls or floors of building constructions to enhance indoor thermal comfort and reduce energy demand for heating and cooling [9,11], used in systems for water heating and cooling [12,13], applied alone or combined with photovoltaic panels for solar thermal energy storage [13,14], employed to build smart thermo-regulating and technical garments [15,16,17], or incorporated into electronic devices to control the temperature and avoid overheating [18,19]. In most of these applications, TES systems are only juxtaposed to the main structure as an additional component. However, in those fields where weight and volume savings are a key issue, such as automotive, aerospace, portable electronics, and technical garments industries, it would be extremely advantageous to embed TES capability directly into the structural mass of the component, thus obtaining a multifunctional structure. In this way, the device would gain TES ability without additional mass or volume, since the structural part would also contribute to the management of thermal energy. To this aim, polymer composites appear to be particularly suitable, since they can be composed of various phases and are easily tailorable to multifunctionality [20,21,22]. Moreover, they exhibit highly specific mechanical properties, since they combine the toughness and the lightness of a polymer matrix with the outstanding stiffness and strength of high-performance fibers [23]. 

Up to now, little has been done to investigate the possibility of designing and fabricating such structural TES composites and to characterize their mechanical and thermal properties [24]. Recently, Yoo et al. [25] added a microencapsulated PCM to a traditional glass fiber–reinforced composite. After a detailed thermal characterization, the authors found that one of the major problem of the system was the debonding between the microcapsules and the matrix, which caused a decrease in the thermal conductivity and possibly also led to an impairment of the mechanical properties, even if the composites were not characterized from the mechanical point of view.

In the present work, a novel polymer matrix has been developed that displays both a good capability of storing and releasing thermal energy and a profile of mechanical properties that make it suitable to fabricate structural composites. This has been achieved by combining a paraffinic PCM, whose confinement was reached with CNTs, with a high-performance epoxy matrix. The selected wax/CNT PCM was added in powder form to an epoxy resin in different weight fractions. The obtained nanocomposite blends were subjected to microstructural, mechanical, and thermal characterization.

## 2. Materials and Methods

### 2.1. Materials

RT44HC^®^ paraffin wax (melting temperature = 44 °C, density at 25 °C = 0.8 g/cm^3^) was supplied by Rubitherm Technologies GmbH (Berlin, Germany) in a metal tank and stored at room temperature. Multi walled carbon nanotubes NC7000^®^ (average diameter 9.5 nm, average length 1.5 µm, BET surface area 250–300 m^2^/g) were provided by Nanocyl SA (Sambreville, Belgium).

The epoxy base Elantech**^®^** EC 157 (density = 1.15 g/ cm^3^, viscosity at 25 °C = 550 mPa·s) and the hardener Elantech^®^ W 342 (density = 0.95 g/cm^3^, viscosity at 25 °C = 50 mPa·s) were supplied by Elantas Europe S.r.l. (Parma, Italy). The hardener consists of a mixture of cycloaliphatic amines. All materials were used as received.

### 2.2. Sample Preparation

#### 2.2.1. Preparation of the Shape-Stabilized PCM (SS-PCM)

In a first preliminary activity (see Supplementary Material Table S1), the paraffin wax was mixed with various kinds of carbon-based nanofillers (such as carbon black, carbon nanotubes, exfoliated graphite nanoplatelets, and expanded graphite) at different concentrations (from 5 to 20 wt %) to assess their capability to confine the wax, i.e., to avoid PCM leakage above its melting temperature. Carbon nanofillers were added to molten paraffin at 70 °C under mechanical stirring at 500 rpm for 30 min. The mixtures were poured in silicon molds and cooled at ambient temperature, to obtain specimens with rectangular cross section (30 × 5 × 2 mm^3^). To evaluate the confinement capability, the resulting specimens were put on an absorbent paper towel and heated in an oven at 80 °C. By visual observation, it was possible to observe that specimens containing CNTs showed by far the lowest leakage compared to the samples containing other carbonaceous nanofillers at the same concentration (see Supplementary Material Table S1). Therefore, CNTs were selected as shape-stabilizing agents for the subsequent experimental activity. Various works can be found in literature in which the shape-stabilizing properties of the confinement agents are studied in a way similar to the one adopted in [26,27,28,29].

**Figure 1:**
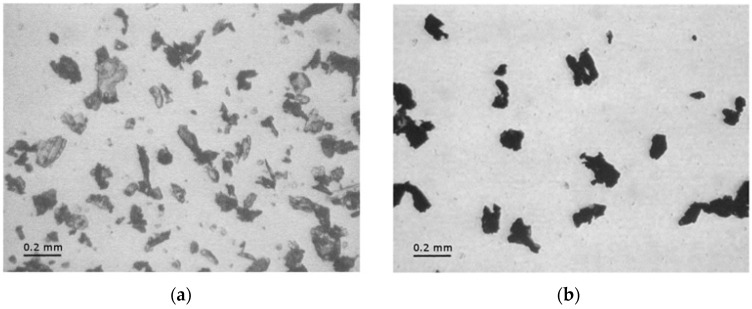
Optical microscope images of (**a**) powder of neat paraffin and (**b**) paraffin-10 wt % CNT.

The optimum weight fraction of CNTs in this application is the minimum amount at which no paraffin leakage is detected above its melting point, since an excess of CNTs would decrease the overall phase change enthalpy of the mixture. Thus, samples with various CNTs amounts (5, 7, and 10 wt %) were prepared as described above and confinement tests were performed. While the samples with 5 and 7 wt % of CNTs showed some leakage of paraffin on the absorbent paper, no leakage was detected for the sample with a CNTs amount of 10 wt %. This CNT concentration was therefore selected as the optimal filler amount for the subsequent process. Both neat and CNT-confined paraffins were ground with a metallic file. Figure 1 shows optical microscope images of the obtained powders, acquired through a Wild Heerbrugg M3Z optical microscope (Heerbrugg, Switzerland) equipped with an Allied Pike F032C camera (Allied Vision Technologies GmbH, Exton, PA, USA). It is possible to observe how, in both cases, micrometric powders with irregular shape are obtained. Through a dimensional analysis with ImageJ^®^ software (NIH, Bethesda, MD, USA), an average powder size of 134 ± 55 µm for the neat paraffin and of 148 ± 52 µm for the CNT-confined paraffin were respectively determined.

#### 2.2.2. Preparation of Epoxy/PCM Blends

The epoxy base and the hardener were mixed at ambient temperature at a weight ratio of 100:30 and magnetically stirred for 15 min. The mixture was then degassed by means of a vacuum pump, and the CNT-confined paraffin was added at three different relative weight fractions (20, 30, and 40 wt %). The mixture was poured in silicon molds, cured at room temperature for 24 h, and post-cured at 80 °C for 10 h. The same procedure was followed to prepare neat epoxy samples. Table 1 lists the prepared samples and their composition.

### 2.3. Testing Techniques

#### 2.3.1. Scanning Electron Microscopy

Scanning electron microscopy (SEM) micrographs of the cryofracture surfaces of all the samples were acquired through a Jeol IT300 scanning electron microscope (Tokyo, Japan), after Pt-Pd sputtering, applying different acceleration voltages.

#### 2.3.2. Thermal Properties

Thermogravimetric analysis (TGA) was performed with a TA Instruments TGA Q5000 IR thermobalance (New Castle, DE, USA). Samples of approximately 10 mg were tested at a heating rate of 10 °C/min up to 700 °C under nitrogen flow. In this way, the degradation temperatures of the various components were investigated and the actual weight fraction of paraffin in the samples EP-ParX and EP-ParX-CNT (X = 20, 30, 40) was calculated from the degradation step of paraffin.

Differential scanning calorimetry (DSC) was performed with a Mettler DSC30 calorimeter (Columbus, OH, USA), at a heating/cooling rate of 10 °C/min, under a nitrogen flow of 100 mL/min. Par and Par-CNTX (X = 5, 7, 10) samples were tested between 0 and 80 °C. EP, EP-ParX and EP-ParX-CNT (X = 20, 30, 40) were tested between −20 and 200 °C. All the specimens underwent a first heating scan, a cooling scan and a second heating scan at the same rate of 10 °C/min. In this way, it was possible to determine the melting and crystallization temperatures ($T_{m}$, $T_{c}$), melting ($\Delta H_{m}$) and crystallization ($\Delta H_{c}$) enthalpies of the paraffin wax, and the glass transition temperature ($T_{g}$) of the epoxy resin. Moreover, relative melting ($\Delta H_{m}^{rel}$) and crystallization ($\Delta H_{c}^{rel}\;$) enthalpies were determined by normalizing $\Delta H_{m}$ and $\Delta H_{c}$ values to the weight fraction of paraffin, as reported in Equation (1):

(1) $$\Delta H_{m}^{rel}=\frac{\Delta H_{m}/w_{p}}{\Delta H_{m}^{neat}};\Delta H_{c}^{rel}=\frac{\Delta H_{c}/w_{p}}{\Delta H_{c}^{neat}}$$

where $w_{p}$ is the weight fraction of paraffin and $\Delta H_{m}^{neat}$ and $\Delta H_{c}^{neat}$ are the specific melting and crystallization enthalpies of neat paraffin, respectively. In addition, the values of melting and crystallization enthalpies were normalized both to the nominal paraffin weight fraction, to obtain $\Delta H_{m}^{rel,n}$ and $\Delta H_{c}^{rel,n}$ values, and to the real paraffin weight fraction as determined from TGA analysis, to obtain $\Delta H_{m}^{rel,r}$ and $\Delta H_{c}^{rel,r}$ values.

Finally, heating/cooling cyclic tests (from 10 to 70 °C, at 10 °C/min, under a nitrogen flow of 100 mL/min) were carried out on the EP-Par40-CNT sample, to evaluate the thermal stability of the material and the repeatability of the endothermic and exothermic transitions.

Dynamic mechanical thermal analysis (DMTA) tests were performed with a TA Q800DMA instrument (New Castle, DE, USA) under single cantilever bending mode. The nominal sample dimensions were 35 × 5 × 2 mm^3^ and the distance between the grips was fixed at 17.5 mm. The samples were heated at 3 °C/min from 0 to 180 °C, and the mechanical strain was applied at a frequency of 1 Hz. The maximum temperature was chosen well above the *T*_g_ range of the epoxy matrix, which is between 88 and 115 °C according to the datasheet of the producer. 

#### 2.3.3. Mechanical Properties

Three-point flexural tests were performed according to ASTM D790-03 standard. Rectangular specimens with nominal dimensions of 10 × 3 × 60 mm^3^ were obtained by casting in a silicon mold. An Instron 4502 universal testing machine (Norwood, MA, USA), equipped with a 1 kN load cell, was used to test specimens at a constant cross-head speed of 1 mm/min and a span length fixed at 40 mm. The tests were conducted until complete fracture of the specimens. At least five specimens were tested for each sample. The tangent modulus of elasticity (E), the flexural strength ($\sigma_{f}$) and the flexural strain at break ($\varepsilon_{f}$) were determined for each specimen according to Equations (2)–(4), respectively, as,

(2) $$E=L^{3}m/4bd^{3}$$

(3) $$\sigma_{f}=3PL/2bd^{2}$$

(4) $$\varepsilon_{f}=6Dd/L^{2}$$

where L is the support span, m is the slope of the tangent to the initial portion of the load-deflection curve, b is the specimen width, d is the specimen thickness, P is the maximum load, and D is the deflection at the break point.

#### 2.3.4. Electrical Properties

The electrical resistivity of Par-CNTX (X = 5, 7, 10) and of EP-ParX-CNT (X = 20, 30, 40) samples was measured in a four-point configuration according to the standard ASTM D4496-04. The measure was performed on the rectangular cast specimens (10 × 3 × 60 mm^3^). A DC voltage generator ISO-Tech IPS 303DD (Milano, Italy) was connected to the specimen, and an ammeter was connected in series to measure the current flowing in the specimen. A voltmeter was connected to the two inner electrodes to measure the voltage drop between them. The inner electrodes were placed at 3.69 mm from each other. The value of electrical resistivity (ρ) was determined at input voltages of 2 and 12 V through Equation (5) as,

(5) ρ=R·(A/t)

where R is the electrical resistance calculated as the ratio between the measured voltage drop and the measured current, A is the cross-sectional area of the specimen, and t is the distance between the two inner electrodes.

## 3. Results and Discussion

### 3.1. Shape-Stabilized PCM (SS-PCM)

#### 3.1.1. Microstructure

**Figure 2:**
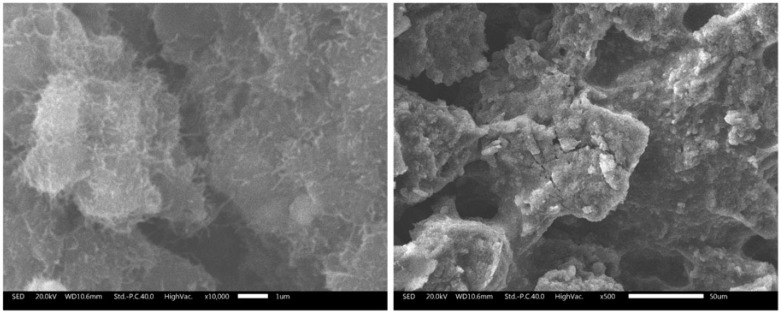
Scanning electron microscopy (SEM) micrographs of Par-CNT10 sample at two different magnifications.

Figure 2 shows SEM micrographs of the cryofractured surface of the Par-CNT10 sample at two different magnifications levels. Single CNTs are clearly visible in the micrograph at higher magnification, and they appear to be well dispersed in the paraffin wax. Their elevated specific surface area is most probably the reason for their superior shape-stabilizing capability with respect to the other carbon nanofillers considered in the preliminary activity. A similar consideration was also reported by Hasnain in a review paper on sustainable thermal energy storage technologies [1].

#### 3.1.2. Thermal Properties

**Figure 3:**
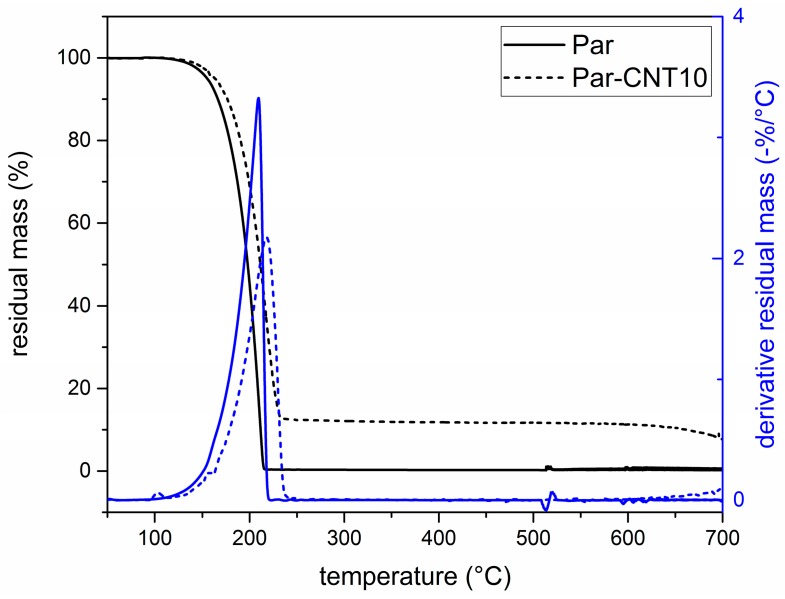
Thermogravimetric analysis (TGA) thermograms of the Par and Par-CNT10 samples.

In Figure 3 the TGA curves of the neat paraffin and the Par-CNT10 sample are reported along with the trends of the residual and the derivative mass as a function of temperature. Thermograms of the Par-CNT5 and Par-CNT7 samples manifested similar features and are not reported for the sake of brevity. The degradation of paraffin occurs in a single step in both samples, and a residual CNT content of 10 wt % is observable in the Par-CNT10 sample. The derivative peak of the degradation process occurs at a higher temperature for Par-CNT10 (218.0 °C) than for neat paraffin (209.4 °C), which is an index of a higher thermal stability.

**Figure 4:**
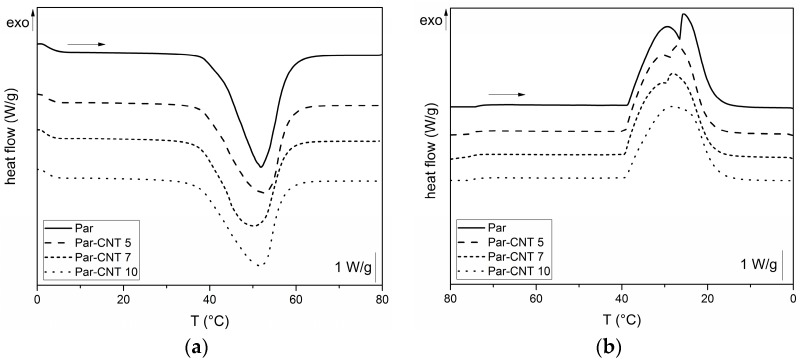
Differential scanning calorimetry (DSC) thermograms of the Par and Par-CNT X samples (X = 5, 7, 10): (**a**) first heating scan and (**b**) cooling scan.

In Figure 4a,b, DSC thermograms of the neat paraffin and of the Par-CNTX (X = 5, 7, 10) samples collected during the first heating and cooling scans are respectively reported.

For all the samples, an endothermic melting peak in the heating scan at approximately 44 °C can be observed, while an exothermic crystallization peak is detected during the cooling phase. The melting window is approximately in the range from 35 to 55 °C, while the crystallization on cooling occurs between 40 and 20 °C. Two local maxima are detectable in the crystallization exothermic region for unfilled paraffin while its intensity is progressively reduced when CNTs are added. This is probably due to the presence of fractions of paraffin having different molecular weight distributions. Table 2 shows the most important results from DSC tests. The specific phase change enthalpy of the neat paraffin is 242.1 J/g, while the enthalpies of the other samples are proportional to the nominal weight fraction of the paraffin (i.e., $\Delta H_{m}^{rel,n}$ and $\Delta H_{c}^{rel,n}$ values are nearly 100%). Thus, no loss of melting/crystallization enthalpy upon CNTs addition can be observed (see Supplementary Material Table S2 for further DSC results). DSC test was also performed on the grinded Par-CNT10 powder. The values of melting and crystallization enthalpies, as well as the shape of the thermogram (not reported for sake of brevity) were quite similar to those measured on the bulk sample with the same composition. Thus, it can be concluded that the grinding process does not affect the thermal behaviour of the PCM.

#### 3.1.3. Electrical Properties

Table 3 reports the values of electrical resistivity of Par-CNTX (X = 5, 7, 10) samples. All the samples present a typical ohmic electrical behaviour in the considered voltage interval, with the electrical resistivity being independent from the applied voltage. As commonly observed in polymer nanocomposites [30,31], CNTs addition determines an increase of the electrical conductivity, proportionally to the nanofiller amount. For instance, with a CNT loading of 10 wt % a resistivity value as low as 32 Ω·cm can be reached. 

### 3.2. Epoxy/SS-PCM Blends

#### 3.2.1. Microstructural Properties

**Figure 5:**
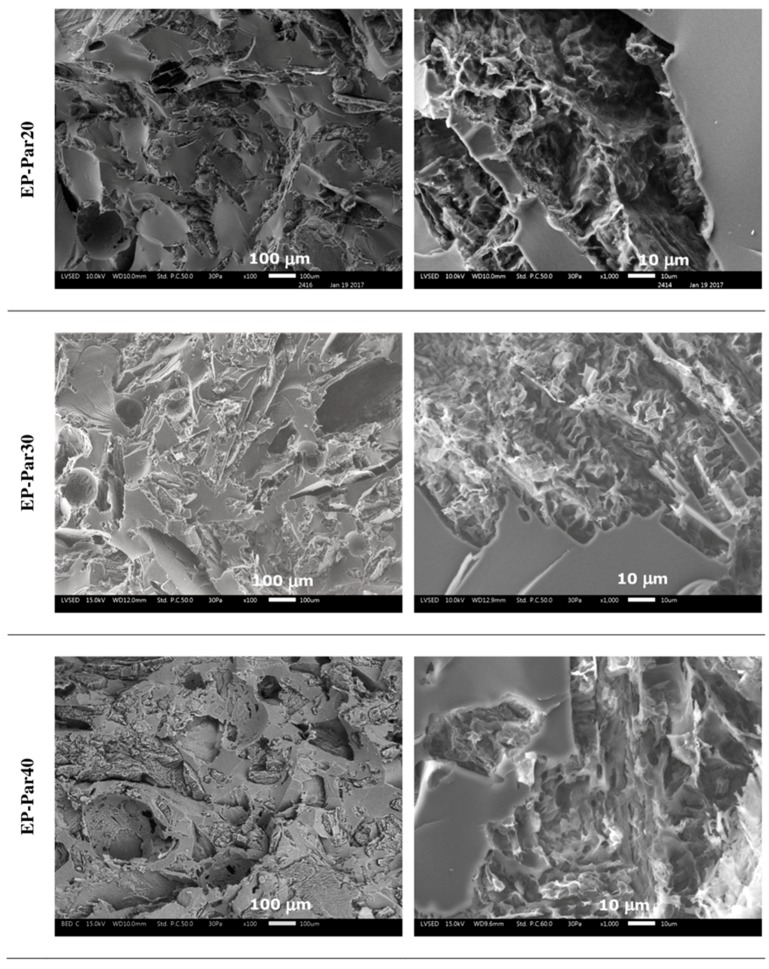
SEM micrographs of the cryofracture surface of EP-ParX samples (X = 20, 30, 40) at two different magnifications.

**Figure 6:**
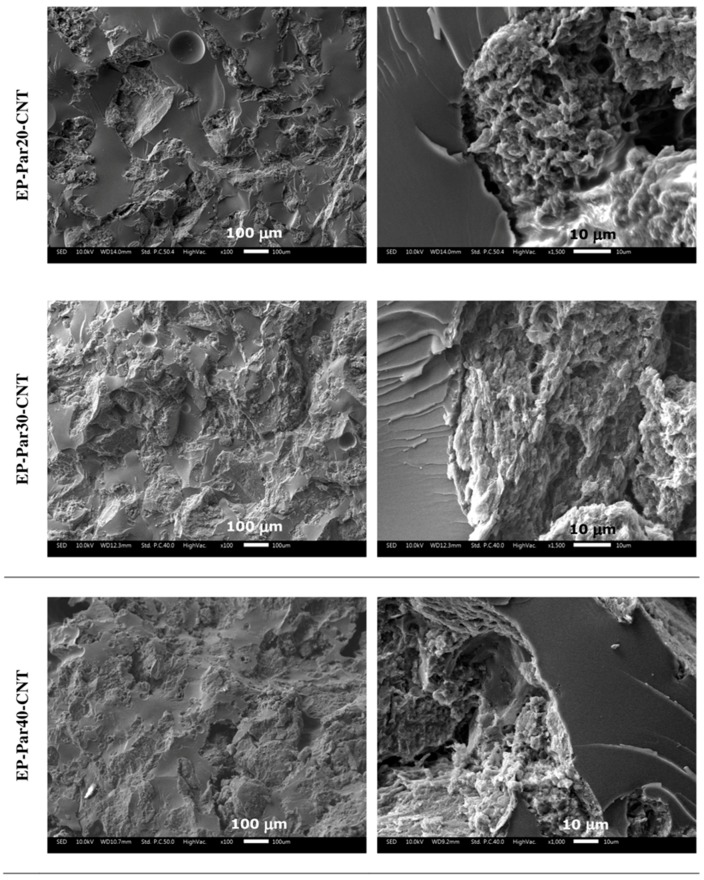
SEM micrographs of the cryofracture surfaces of the EP-ParX-CNT samples (X = 20, 30, 40) at two different magnifications.

SEM micrographs of the samples obtained by combining epoxy resin with neat paraffin and with Par-CNT10 powder are reported in Figure 5 and Figure 6, respectively. In the micrographs at low magnification, it can be seen that the paraffin particles still show an irregular shape and a rough surface topography, while the epoxy matrix shows a smooth profile, due to its brittle fracture behaviour. In the samples with the highest PCM content (i.e., EP-Par40 and EP-Par40-CNT samples), the PCM and the epoxy resin assume a co-continuous structure. 

Some pores can be noticed in all samples, which are due to the air entrapped during processing. In the micrographs at high magnification, the interface between the two phases can be better appreciated. Some interfacial debonding phenomena are visible in the EP-ParX samples (Figure 5), while the interfacial adhesion seems to be improved in EP-ParX-CNT samples by the presence of CNTs (Figure 6). The enhanced interfacial adhesion between the epoxy and the PCM phases due to the presence of CNTs could influence the failure mechanisms of the resulting blends.

#### 3.2.2. Thermal Properties

**Figure 7:**
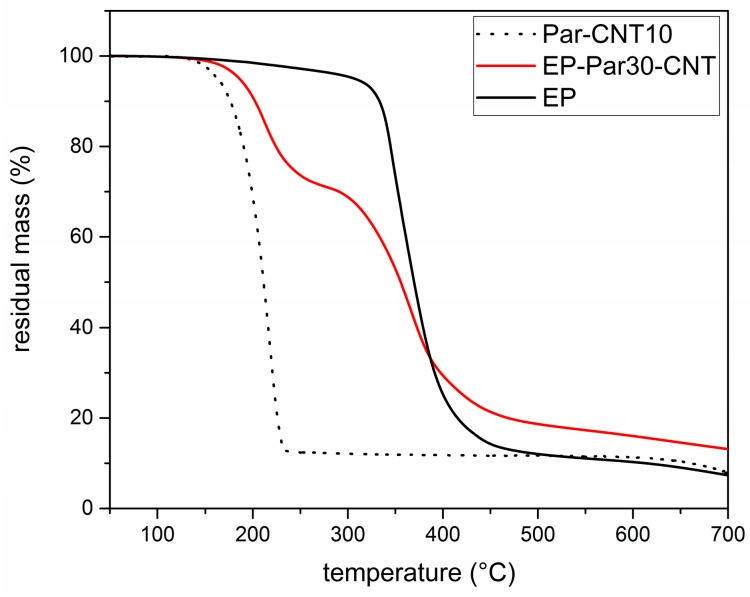
TGA thermograms of the Par-CNT10, EP, and EP-Par30-CNT samples.

Figure 7 shows the TGA thermograms of Par-CNT10, the neat epoxy resin (EP), and the sample EP-Par30-CNT, reported as an example of the behaviour of the samples EP-ParX and EP-ParX-CNT (X = 20, 30, 40) (the whole set of thermograms is reported in Supplementary Material Figure S1 and Table S3). The degradation of EP-Par30-CNT occurs in two distinct steps, which can be associated to the degradation of the paraffin and the epoxy resin, respectively. From the degradation step of the paraffin, and considering the mass loss from the epoxy resin fraction at that temperature, it is possible to estimate the actual paraffin amount within the sample. Table 4 shows the nominal and real paraffin fraction after processing for each sample (see Supplementary Material Figure S1 and Table S3 for more detailed TGA results).

These two values are approximately the same for each EP-ParX-CNT (X = 20, 30, 40) sample, which is an indication that the method adopted for calculating the mass is reliable. In contrast, for the three samples without CNTs, the real paraffin fraction after processing is noticeably lower than the nominal content (tests were repeated to assess sample homogeneity). This is probably due to the partial exudation of the paraffin out of the epoxy matrix during the curing step at 100 °C. In fact, EP-ParX (X = 20, 30, 40) specimens became greasy after that curing step because of the presence of a thin paraffin layer on the upper surface of those specimens. None of these phenomena were observed on the EP-ParX-CNT (X = 20, 30, 40) samples, which suggests that an effective shape-stabilization is performed by the CNTs. In a parallel experiment, it was observed that a phase separation phenomenon can be clearly detected if the curing process of the epoxy resin is entirely carried out above the melting temperature of paraffin (i.e., 10 h at 100 °C). In this case, the paraffin melts and moves toward the upper surface of the specimens before the crosslinking process starts, and two well separated layers of paraffin and fully cured epoxy resin are obtained after curing. By performing the initial phase of the curing cycle at room temperature, below the melting temperature of the paraffin, the phase separation is less evident, probably because paraffin is in the form of solid particles and the epoxy-hardener mixture becomes more and more viscous. However, after 24 h at room temperature, the epoxy resin is not fully crosslinked, and a certain phase separation is still possible. The loss of PCM during the process is also reflected in DSC results.

**Figure 8:**
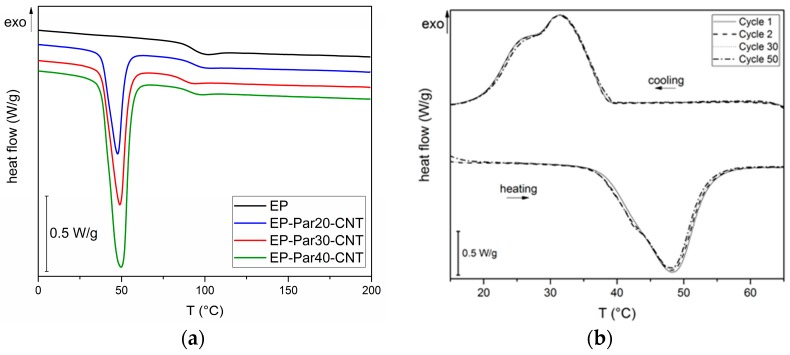
(**a**) DSC thermograms of EP and EP-ParX-CNT samples (X = 20, 30, 40) (first heating scan). (**b**) DSC thermograms of EP and EP-ParX-CNT samples at different heating/cooling cycles.

In Figure 8a, representative thermograms of EP-ParX-CNT (X = 20, 30, 40) samples during the first heating scan are reported. In Table 5, the most important results are summarized.

The glass transition temperature (*T*_g_) of the epoxy is not substantially affected by the presence of paraffin, since the values of *T*_g_ of the EP-ParX and EP-ParX-CNT samples (X = 20, 30, 40) are not significantly different from that found for the neat epoxy resin, which is approximately 92.2 °C. Also, both the melting and crystallization temperatures and intervals of the paraffin are not affected by the presence of the epoxy resin. On the other hand, the effect of the blending process on the melting and crystallization enthalpies can be evaluated by considering the values of relative melting (and crystallization) enthalpies with respect to the nominal paraffin content $\Delta H_{m}^{rel,n}\;$($\Delta H_{c}^{rel,n}),$ and to the real paraffin content calculated from TGA tests $\Delta H_{m}^{rel,r}$ $(\Delta H_{c}^{rel,r}).$ For the samples containing neat paraffin (EP-ParX), $\Delta H_{m}^{rel,n}$ and $\Delta H_{c}^{rel,n}$ values are in the range 50–60%, while for the blends with shape-stabilized paraffin (Ep-ParX-CNT), $\Delta H_{m}^{rel,n}$ and $\Delta H_{c}^{rel,n}$ values around 80–85% can be obtained. Similar results were found in literature for other polymer/organic PCM blends [33]. However, if the enthalpy values are normalized to real paraffin content, all the samples show $\Delta H_{m}^{rel,r}$ and $\Delta H_{c}^{rel,r}$ values close to 80–90%, without any remarkable differences between samples with and without CNTs. These results indicate that the fraction of paraffin contained in the samples is able to melt and crystallize regardless of the presence of CNTs, at least after few thermal cycles. On the other hand, the presence of CNTs is important to prevent exudation of PCM during the production process. As suggested in other studies [27], multiple thermal cycles are required to further investigate the exudation of paraffin in service conditions and to highlight possible differences between the samples with and without CNTs. Relative melting enthalpy values in the range 80–90% are commonly found in literature for paraffin-based phase change materials embedded in a polymeric matrix [27,33]. One of the most common explanations is a non-homogeneous dispersion of the PCM in the matrix [27], but it is probably not the case of this work, since TGA and DSC analyses on multiple specimens of the same sample gave consistent results. Other possible explanations involve the paraffin macromolecules confinement played by the presence of the surrounding matrix, or the partial dissolution in the matrix of a fraction of paraffin [33]. However, further tests will be needed to fully understand this aspect.

Lastly, to evaluate the retention of the thermal energy storage/release effect over repeated thermal cycles, cyclic DSC tests were performed on the EP-Par40-CNT sample. In Figure 8b DSC thermograms during the 1st, 2nd, 30th, and 50th cycles are reported. The thermograms at different cycles are nearly superimposed, and there are no appreciable differences between the phase change temperatures and enthalpies of the sample at different cycles. It can be concluded that the melting and crystallization enthalpy of the epoxy/SS-PCM blends are stable up to 50 cycles in the analyzed temperature range. These results are promising for the future development of a thermally reliable multifunctional composite material.

**Figure 9:**
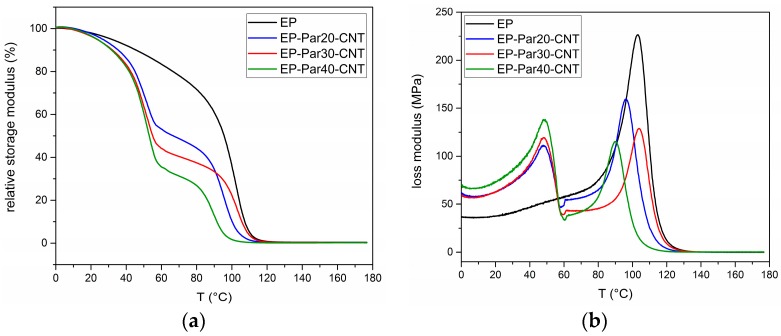
(**a**) Relative storage modulus as a function of temperature of the samples EP and EP-ParX-CNT (X = 20, 30, 40). (**b**) Loss modulus as a function of temperature of the samples EP and EP-ParX-CNT (X = 20, 30, 40).

Figure 9a,b shows the main results of DMTA tests. The relative storage modulus (E′) (respect to the value at 0 °C) as a function of temperature of the samples EP and EP-ParX-CNT (X = 20, 30, 40) is presented in Figure 9a.

All the samples present a sharp decrease of E′ between 85 and 100 °C, in correspondence of the glass transition of the epoxy resin. Additionally, the blends EP-ParX-CNT (X = 20, 30, 40) show a decrease of E′ at approximately 50 °C, in correspondence of the melting of the paraffin. In the same way, the loss modulus of all the samples in Figure 9b show peaks between 85 and 100 °C, while in the samples EP-ParX-CNT (X = 20, 30, 40) the melting of the paraffin is indicated by the peaks of the loss modulus at around 50 °C (see Supplementary Materials Figure S2 and Table S4 for additional information on DMTA results).

#### 3.2.3. Mechanical Properties

**Figure 10:**
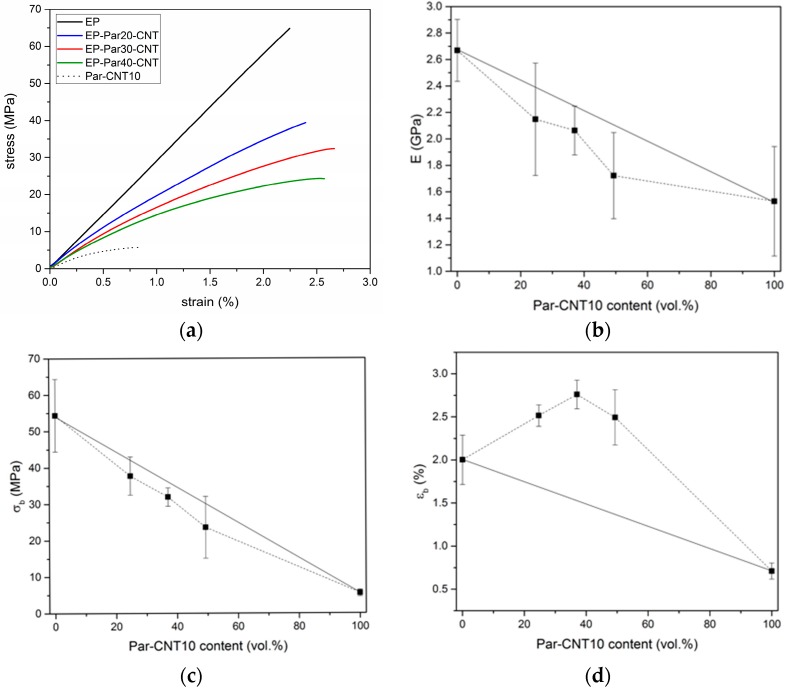
(**a**) Representative stress-strain curves from flexural tests on the EP sample, EP-ParX-CNT samples (X = 20, 30, 40) and Par-CNT 10 samples. The stress-strain curves end with the final rupture of the specimens, where the load drops suddenly to zero. Trends of (**b**) flexural modulus, (**c**) flexural stress at break, and (**d**) flexural strain at break as a function of the Par-CNT10 volume fraction.

Figure 10a shows representative stress-strain curves obtained under three-point flexural configuration on neat epoxy, Par-CNT10, and EP-ParX-CNT blends (X = 20, 30, 40). EP sample shows a linear elastic behaviour, while the curves of the Par-CNT10 and of the blends deviate from linearity already at low strain levels. Table 6 summarizes the values of the most important mechanical properties.

The flexural modulus (E) of neat epoxy results as 2.66 GPa and it decreases with an increase of the paraffin content. The same trend can be found for the stress at break ($\sigma_{f}$). The values of strain at break ($\varepsilon_{f}$) of the EP-ParX-CNT blends are larger than those of EP and Par-CNT10 samples. The same effects can also be observed for the EP-ParX blends. The trends of the flexural properties as a function of the PCM relative amount are reported in Figure 10b–d. The values of modulus and stress at break of the blends EP-ParX-CNT vary almost linearly with the volume fraction of Par-CNT10, following the rule of mixtures. On the other hand, the values of strain at break show a positive deviation from the linear trend thus suggesting a synergistic interaction between the components of the blend. This result could be tentatively explained by the presence of some paraffin in the crosslinked network of the epoxy resin, which acts as a plasticizer within the material, but further efforts will be devoted to fully understand the phenomenon. The decrease in E and $\sigma_{f}$ was less evident in the samples containing CNTs, and the observed reduction of the mechanical properties is acceptable for a possible application of these materials as matrices for multifunctional structural composites reinforced with continuous high-strength fibers.

#### 3.2.4. Electrical Properties

Electrical resistivity values of the EP-ParX CNT blends are shown in Table 7. As observed for Par-CNTX samples (see Section 3.1.3), the electrical resistivity does not depend on the applied voltage and it decreases with an increase of the CNT weight fraction. An electrical resistivity value of 1.2 × 10^3^ Ω·cm can be detected for the EP-Par40-CNT sample. This means that the low electrical resistivity observed for the Par-CNT10 sample (i.e., 1.5 × 10^2^ Ω·cm) allows the development of electrically conductive multifunctional composites with thermal energy storage capability.

## 4. Conclusions

In this work, an epoxy matrix was blended with a CNT-confined paraffin at different concentrations, and the microstructure and physical properties of the resulting materials were investigated. As a preliminary step, paraffin containing a CNT weight fraction of 10 wt % was ground to obtain a powder, and DSC analysis showed that CNT addition did not noticeably decrease the melting and crystallization enthalpy with respect to the pristine paraffin wax. The obtained shape-stabilized PCM powder was added to the epoxy resin at different concentrations. DSC tests showed that the paraffin was able to melt and crystallize also in the blends, with elevated relative melting and crystallization enthalpy values (80–90%), similar to those obtained for other organic PCM blends [33], and the blending process did not modify the melting and crystallization peak temperatures and intervals. On the other hand, the noticeable melting enthalpy loss observed for epoxy/PCM blends without CNTs highlighted the effectiveness of the CNTs as a shape-stabilizing agent and their importance in preventing the paraffin exudation out of the epoxy matrix, as evidenced by TGA tests. The thermal reliability of the prepared blends was assessed through cyclic DSC tests, and a substantial retention of the thermal properties was observed even after 50 thermal cycles. The presence of paraffin lowers the elastic modulus and flexural strength of the epoxy matrix with a trend in accordance with the mixture rule. In contrast, flexural strain at break values deviate positively from the theoretical linear trend. Moreover, CNTs introduction allowed the preparation of epoxy/PCM blends with interesting electrical properties. The results presented in this study show potential for the development of a structural TES composite, since this new matrix could be used to build a multifunctional composite with the introduction of high-performance fibers, which will be the focus of upcoming studies.

## Figures and Tables

**Table 1:** List of the samples investigated in this work.

Sample Code	Epoxy Resin Content (wt %)	Paraffin Content (wt %)	CNTs Content (wt %)
Par	0	100	0
EP	100	0	0
Par-CNT 5	0	95	5
Par-CNT 7	0	93	7
Par-CNT 10	0	90	10
EP-Par 20	80	20	0
EP-Par 30	70	30	0
EP-Par 40	60	40	0
EP-Par 20-CNT	80	18	2
EP-Par 30-CNT	70	27	3
EP-Par 40-CNT	60	36	4

**Table 2:** DSC results of the Par and Par-CNT X samples (X = 5, 7, 10).

Sample	$T_{m}$ (°C)	$\Delta H_{m}$ (J/g)	$\Delta H_{m}^{rel,n}$ (%)	$T_{c}$ (°C)	$\Delta H_{c}$ (J/g)	$\Delta H_{c}^{rel,n}\;$(%)
Par	44.5	242.1	100.0	32.0	241.2	100.0
Par-CNT 5	46.7	235.6	102.2	33.2	233.3	101.8
Par-CNT 7	45.3	231.7	102.6	33.1	228.5	101.7
Par-CNT 10	46.4	219.1	100.6	32.4	215.5	99.1

$T_{m}$ = melting temperature $\Delta H_{m}$ = meting enthalpy $\Delta H_{m}^{rel,n}$ = melting enthalpy relative to the to the nominal paraffin fraction $T_{c}$ = crystallization peak temperature $\Delta H_{c}$ = crystallization enthalpy $\Delta H_{c}^{rel,n}$ *=* crystallization enthalpy relative to the nominal paraffin fraction.

**Table 3:** Electrical resistivity of the Par-CNT X samples (X = 5, 7, 10).

Sample	Resistivity (Ω·cm)
Par	10^19^ *
Par-CNT 5	3.3 × 10^2^
Par-CNT 7	1.5 × 10^2^
Par-CNT 10	3.2 × 10^1^

* literature value [32].

**Table 4:** Nominal and real paraffin content after processing of the samples EP-ParX and EP-ParX-CNT (X = 20, 30, 40) samples as determined from TGA tests.

Sample	Nominal Paraffin Content (%)	Real Paraffin Content (%)
EP-Par20	20	14.04
EP-Par30	30	19.62
EP-Par40	40	24.23
EP-Par20-CNT	18	17.49
EP-Par30-CNT	27	27.24
EP-Par40-CNT	36	35.67

**Table 5:** DSC results of the EP-ParX and EP-ParX-CNT samples (X = 20, 30, 40).

Sample	$T_{g}$ (°C)	$T_{m}$ (°C)	$\Delta H_{m}$ (J/g)	$\Delta H_{m}^{rel,n}$ (%)	$\Delta H_{m}^{rel,r}$ (%)	$T_{c}$ (°C)	$\Delta H_{c}$ (J/g)	$\Delta H_{c}^{rel,n}$ (%)	$\Delta H_{c}^{rel,r}$ (%)
Par	-	44.5	242.1	100.00	100.0	35.0	241.2	100.0	100.0
EP-Par20	92.1	45.0	26.6	55.02	78.4	34.8	24.6	50.9	72.6
EP-Par30	92.0	48.6	42.0	57.87	88.5	31.3	39.4	54.5	83.3
EP-Par40	96.1	46.7	52.6	54.32	89.7	32.8	49.0	50.8	83.9
EP-Par20-CNT	92.7	46.2	37.1	85.13	87.6	33.6	33.8	77.9	80.1
EP-Par30-CNT	91.8	47.1	56.2	85.98	85.2	32.5	53.1	81.5	80.8
EP-Par40-CNT	89.3	47.6	71.4	81.92	82.7	32.3	70.7	81.4	82.2
EP	92.2	-	-	-	-	-	-	-	-

$T_{g}$ = glass transition temperature of the epoxy resin $T_{m}$ = melting temperature $\Delta H_{m}$ = melting enthalpy $\Delta H_{m}^{rel,n}$ = melting enthalpy relative to the nominal paraffin content $\Delta H_{m}^{rel,r}$ = melting enthalpy relative to the real paraffin content $T_{c}$ = crystallization temperature $\Delta H_{c}$ = crystallization enthalpy $\Delta H_{c}^{rel,n}$ = crystallization enthalpy relative to the nominal paraffin content $\Delta H_{c}^{rel,r}$ = crystallization enthalpy relative to the real paraffin content.

**Table 6:** Flexural properties of the EP, the EP-ParX samples, and the EP-ParX-CNT samples (X = 20, 30, 40).

Sample	*E* (GPa)	σ_f_ (MPa)	ε_f_ (%)
EP	2.66 ± 0.23	53 ± 9	2.00 ± 0.28
EP-Par20-CNT	2.14 ± 0.14	38 ± 5	2.52 ± 0.12
EP-Par30-CNT	2.06 ± 0.18	32 ± 2	2.76 ± 0.16
EP-Par40-CNT	1.72 ± 0.32	24 ± 8	2.49 ± 0.32
EP-Par20	1.40 ± 0.12	35 ± 4	2.78 ± 0.19
EP-Par30	0.99 ± 0.07	26 ± 2	2.93 ± 0.23
EP-Par40	0.67 ± 0.08	16 ± 1	3.09 ± 0.15
Par-CNT 10	1.53 ± 0.41	5.5 ± 0.9	0.71 ± 0.09

**Table 7:** Electrical resistivity of the EP-ParX-CNT samples (X = 20, 30, 40).

Sample	Resistivity (Ω·cm)
EP-Par20-CNT	5.7 × 10^4^
EP-Par30-CNT	5.5 × 10^3^
EP-Par40-CNT	1.2 × 10^3^

## Supplementary Materials

The following are available online at www.mdpi.com/2073-4360/9/9/405/s1, Figure S1: TGA thermograms of (a) the EP-ParX samples (X = 20, 30, 40), compared with the thermograms of the neat epoxy (EP) and the neat paraffin (Par) and (b) the EP-ParX-CNT samples (X = 20, 30, 40), compared with the thermograms of the neat epoxy (EP) and the Par-CNT 10 sample. Figure S2: DMTA tests. Tanδ as a function of temperature of the samples EP and EP-ParX-CNT (X = 20, 30, 40). Table S1: Qualitative assessment of the shape-stabilizing performance of different carbon micro- and nanofillers. Samples were prepared in the same way as described in the main text and kept for 30 min at 80 °C, to observe possible leakage. Table S2: DSC results of the onset and endset temperatures of the first heating and cooling scan on the Par and Par-CNT X samples (X = 5, 7, 10). The higher crystallization onset temperature of the filled samples with respect to that of the neat paraffin may indicate a nucleation effect due to the presence of carbon nanotubes. Table S3: data obtained from TGA thermograms of Figure S1. First and second dTG peaks are due to the degradation of the paraffin wax and the epoxy resin, respectively. Table S4: DMTA data. Values of storage modulus below all the transitions (0 °C), between the melting temperature of the paraffin and the *T*_g_ of the epoxy resin (70 °C) and above the melting temperature of the epoxy resin (140 °C). Two peak temperatures in the trend of the loss modulus were detected, which are associated to the melting of paraffin and the glass transition of the epoxy resin, respectively.
