# Supplementary material for: Wax Confinement with Carbon Nanotubes for Phase Changing Epoxy Blends
Source: Polymers (Basel). 2017 Aug 31;9(9):405. doi: 10.3390/polym9090405 (PMC6418604; doi:10.3390/polym9090405)
Supplement: Supplementary file 1 [file polymers-09-00405-s001.docx]

**Supplementary material**





**(a)**





**(b)**

**Figure S1:** TGA thermograms of **(a)** the EP-ParX samples (X = 20, 30, 40), compared with the thermograms of the neat epoxy (EP) and the neat paraffin (Par) and **(b)** the EP-ParX-CNT samples (X = 20, 30, 40), compared with the thermograms of the neat epoxy (EP) and the Par-CNT 10 sample.





Figure S2: DMA tests. Tanδ as a function of temperature of the samples EP and EP-ParX-CNT (X = 20, 30, 40).

**Table S1**: Qualitative assessment of the shape‑stabilizing performance of different carbon micro- and nanofillers. Samples were prepared in the same way as described in the main text and kept for 30 minutes at 80°C, to observe possible leakage.

| Sample | Filler | Filler wt% | Leakage detected (Y/N) |
| --- | --- | --- | --- |
| Par-CNT 5 | carbon nanotubes^1^ | 5 | Y |
| Par-CNT 7 | carbon nanotubes^1^ | 7 | Y |
| Par-CNT 10 | carbon nanotubes^1^ | 10 | N |
| Par-CNT 15 | carbon nanotubes^1^ | 15 | N |
| Par-xGnP 5 | expanded graphite nanoplatelets^2^ | 5 | Y |
| Par-xGnP 10 | expanded graphite nanoplatelets^2^ | 10 | Y |
| Par-xGnP 15 | expanded graphite nanoplatelets^2^ | 15 | N |
| Par-EG 5 | expanded graphite^3^ | 5 | Y |
| Par-EG 10 | expanded graphite^3^ | 10 | Y/N |
| Par-EG 15 | expanded graphite^3^ | 15 | N |
| Par-CB 5 | carbon black^4^ | 5 | Y |
| Par-CB 10 | carbon black^4^ | 10 | Y |

^1^ Carbon nanotubes Nanocyl 7000 (described in the main text)

^2^ xGnP grade M, XGScience

^3^ Expandable graphite ES 250 B5, Qingdao Kropfmuehl, expanded in an oven at 800°C

^4^ Carbon black Ketjenblack EC600JD, Lion Specialty Chemicals Co. Ltd.

**Table S2**: DSC results of the onset and endset temperatures of the first heating and cooling scan on the Par and Par-CNT X samples (X = 5, 7, 10). The higher crystallization onset temperature of the filled samples with respect to that of the neat paraffin may indicate a nucleation effect due to the presence of carbon nanotubes.

| Sample | $\boldsymbol{T}_{\boldsymbol{m}}^{\boldsymbol{onset}}$  (°C) | $\boldsymbol{T}_{\boldsymbol{m}}^{\boldsymbol{endset}}$  (°C) | $\boldsymbol{T}_{\boldsymbol{c}}^{\boldsymbol{onset}}$  (°C) | $\boldsymbol{T}_{\boldsymbol{c}}^{\boldsymbol{endset}}$  (°C) |
| --- | --- | --- | --- | --- |
| Par | 39.06 | 53.72 | 38.30 | 22.82 |
| Par-CNT 5 | 38.12 | 56.48 | 38.88 | 21.32 |
| Par-CNT 7 | 38.17 | 56.53 | 40.01 | 20.13 |
| Par-CNT 10 | 36.71 | 55.42 | 39.77 | 19.34 |

**Table S3**: data obtained from TGA thermograms of Figure S1. First and second dTG peaks are due to the degradation of the paraffin wax and the epoxy resin, respectively.

| Sample | Mass loss at 260°C  (%) | Residual mass at 700°C  (%) | 1^st^ dTG peak  (°C) | 2^nd^ dTG peak  (°C) |
| --- | --- | --- | --- | --- |
| EP | 3.366 | 9.10 | -- | 347.77 |
| Par | 99.70 | 0.21 | 209.38 | -- |
| Par-CNT-10 | 89.13 | 9.56 | 218.05 | -- |
| EP-Par 20 | 16.79 | 9.60 | 194.21 | 368.19 |
| EP-Par 30 | 22.27 | 10.90 | 198.23 | 367.96 |
| EP-Par 40 | 26.53 | 11.32 | 201.55 | 364.07 |
| EP-Par 20-CNT | 19.85 | 11.40 | 199.68 | 366.00 |
| EP-Par 30-CNT | 28.99 | 13.14 | 212.31 | 366.83 |
| EP-Par 40-CNT | 36.51 | 13.62 | 217.65 | 364.52 |

**Table S4:** DMTA data. Values of storage modulus below all the transitions (0°C), between the melting temperature of the paraffin and the Tg of the epoxy resin (70°C) and above the melting temperature of the epoxy resin (140°C). Two peak temperatures in the trend of the loss modulus were detected, which are associated to the melting of paraffin and the glass transition of the epoxy resin, respectively.

| Sample | $\boldsymbol{E}^{\boldsymbol{'}}$at 0°C  (MPa) | $\boldsymbol{E}^{\boldsymbol{'}}$at 70°C  (MPa) | $\boldsymbol{E}^{\boldsymbol{'}}$at 140°C  (MPa) | $\boldsymbol{E}^{\boldsymbol{''}}\boldsymbol{,}$1^st^ peak  (°C) | $\boldsymbol{E}^{\boldsymbol{''}}\boldsymbol{,}$2^nd^ peak  (°C) |
| --- | --- | --- | --- | --- | --- |
| EP | 2575.2 | 2009.0 | 10.0 | -- | 103.38 |
| EP-Par 20-CNT | 2569.4 | 1251.6 | 5.7 | 47.79 | 96.36 |
| EP-Par 30-CNT | 2440.3 | 983.0 | 4.23 | 48.56 | 104.14 |
| EP-Par 40-CNT | 2641.4 | 819.3 | 3.08 | 49.08 | 90.64 |
|  |  |  |  |  |  |
